# Supplementary material for: Simultaneous Increase in CO2 and Temperature Alters Wheat Growth and Aphid Performance Differently Depending on Virus Infection
Source: Insects. 2020 Jul 22;11(8):459. doi: 10.3390/insects11080459 (PMC7469198; doi:10.3390/insects11080459)
Supplement: Supplementary file 1 [file insects-11-00459-s001.pdf]

Supplementary information

Article

Simultaneous elevation of CO_2 and temperature alters wheat growth and aphid performance differently depending on virus infection

Ana Moreno-Delafuente[1,2], Elisa Viñuela[1,4], Alberto Fereres[3,4], Pilar Medina[1,4], Piotr Trębicki[2*]

[1] Escuela Técnica Superior de Ingeniería Agronómica, Alimentaria y de Biosistemas, Universidad Politécnica de Madrid (ETSIAAB-UPM), Avd. Puerta de Hierro 2-4, Madrid, 28040, Spain; ana.moreno@upm.es (A.M.); elisa.vinuela@upm.es (E.V.); pilar.medina@upm.es (P.M.)

[2] Agriculture Victoria Research, Department of Jobs, Precincts and Regions, 110 Natimuk Rd, Horsham, VIC, 3400, Australia

[3] Instituto de Ciencias Agrarias, Consejo Superior de Investigaciones Científicas (ICA-CSIC), C/ Serrano 115 dpdo., Madrid, 28006, Spain; a.fereres@csic.es (A.F.);

[4] Associate Unit IVAS (CSIC-UPM): Control of Insect Vectors of Viruses in Horticultural Sustainable Systems, Madrid, Spain

* Correspondence: piotr.trebicki@agriculture.vic.gov.au; Tel.: +61-(0)3-4344-3111 (P.T.)

Table S1. Development time of pre-reproductive stages of *Rhopalosiphum padi* (mean values) reared on non-infected or BYDV-PAV infected plants under ambient CO_2&T (aCO_2&aT = 400 ppm; 20 °C) or elevated CO_2&T (eCO_2&eT = 800 ppm; 22 °C).

Aphid parameter	CO_2&T	Virus infection		Mean	SEM	*P*-value		
		Non-infected	BYDV-PAV infected			CO_2&T	virus	CO_2&T x virus
N1 duration (days)	aCO_2&aT	1.000	1.000	1.000	0.036	0.167	1.000	1.000
	eCO_2&eT	1.050	1.050	1.050				
	Mean	1.025	1.025					
N2 duration (days)	aCO_2&aT	1.550	1.684	1.617 A	0.097	**<0.001******	**0.002****	0.064
	eCO_2&eT	1.000	1.500	1.250 B				
	Mean	1.275 b	1.592 a					
N3 duration (days)	aCO_2&aT	1.450	1.316	1.383	0.113	0.710	0.416	0.710
	eCO_2&eT	1.450	1.400	1.425				
	Mean	1.450	1.358					
N4 duration (days)	aCO_2&aT	2.250	2.474	2.362	0.098	0.061	0.167	0.379
	eCO_2&eT	2.150	2.200	2.175				
	Mean	2.200	2.337					
From the beginning of adulthood to the onset of reproduction (days)	aCO_2&aT	2.050	2.105	2.078 B	0.107	**0.033***	0.774	0.572
	eCO_2&eT	2.350	2.250	2.300 A				
	Mean	2.200	2.178					

N1 (first nymphal instar), N2 (second nymphal instar), N3 (third nymphal instar), N4 (forth nymphal instar). *P*-values according to Two-way ANOVA test for normal and homoscedastic variables. "From the beginning of adulthood to the onset of reproduction " parameter was transformed by log (x+1). Significant differences represented by asterisks: * ($P \leq 0.05$), ** ($P \leq 0.01$) and *** ($P \leq 0.001$). Different lower case letters within row indicate differences due to virus infection ($P \leq 0.05$). Different upper case letters within column indicate differences due to CO_2&T conditions ($P \leq 0.05$). SEM: Standard error of means. n = 20.
